# The Weapons of Death

**Published:** 1873-07

**Authors:** 


### THE WEAPONS OF DEATH.

When we reflect how easily life is sometimes lost, and the numberless agencies for its extinction, it almost seems a marvel that any one should live fifty years. It is literally true everywhere that "in the midst of life we are in death."

> "Our life contains a thousand springs
> And dies if one be gone!
> Strange, that a harp of thousand strings
> Should keep in tune so long."

How, then, ought we all to "walk softly," humbly, not knowing "what a day may bring forth." In a late official report of the causes of death in Great Britain it is stated that one man died from the bite of a cat, and two more from the bites respectively of a ferret and an adder. Another was stung to death by bees. A man and a boy died of falling from velocipedes, and an old lady was killed by injuries inflicted by that agreeable machine. The swallowing of a shell, a screw and a cherry stone put a period to the lives of three infants; while two died of putting, one a stone, the other a bead into the ear. Swallowing bones sent three people out of the world, swallowing coins finished two, and swallowing a pin quickly pricked on grim death for one. A scratch from a thorn killed a woman of middle age; improper medicine poisoned eight people, and improper food five; 444 young children were smothered by bed-clothes, and 930 persons during the year lost their lives in railway accidents. The proportion of suicides to every million of the population is about seventy—the deaths by hanging, the knife and drowning being most numerous. Heart disease the year's record shows to be increasing—a state of things which is said by eminent physicians to be caused by the greater wear and tear of business and the increased mental activity of the age.

The Reason.—We have just learned the reason—could not find it out ourselves—why a cat three months and three weeks old crosses the street. As the information may be of service to our readers, we will tell it to them as it was told to us, not vouching for the truth of it, because it is a bare assertion, without any argument or testimony of the fact: It is to get on the other side.

An Emetic.—"Mr. 'Potecary, mother wants you to send her a emetic, and, if it ain't a good one, says she'll send it back and get another."

Spelling.—"How do you think little Mary spells cat?" said a lady to a Doctor of Divinity. "If you can guess in five trials we'll get up a donation party, and pay it all in Irish potatoes, as they did down East last winter." Forthwith the Dominie put on his studying cap, and essayed vehemently:

"K-a-t, cat?" "No."

"K-a-double-tee, cat?" "No."

"C-h-a-t, cat?" "No."

"K-a-h-t, cat?" "No."

"K-a-g-h-t, cat?" "No."

"Well, I don't believe she has sense enough to spell cat at all."

"O yes she has, for she said 'C-a-t spells cat.'"

